# Role of community-based active case finding in screening tuberculosis in Yunnan province of China

**DOI:** 10.1186/s40249-019-0602-0

**Published:** 2019-10-29

**Authors:** Jin-Ou Chen, Yu-Bing Qiu, Zulma Vanessa Rueda, Jing-Long Hou, Kun-Yun Lu, Liu-Ping Chen, Wei-Wei Su, Li Huang, Fei Zhao, Tao Li, Lin Xu

**Affiliations:** 1 Division of tuberculosis control and prevention, Yunnan Center for Disease Control and Prevention, Kunming, China; 2 Universidad Pontificia Bolivariana, Medellín, Colombia; 3 Clinical trail and research center of Beijing hospital, Beijing, China; 4 Chinese Center for Disease Control and Prevention, Beijing, China

**Keywords:** Tuberculosis, Active case finding, Patient delay, Passive case finding, Diagnosis

## Abstract

**Background:**

The barriers to access diagnosis and receive treatment, in addition to insufficient case identification and reporting, lead to tuberculosis (TB) spreads in communities, especially among hard-to-reach populations. This study evaluated a community-based active case finding (ACF) strategy for the detection of tuberculosis cases among high-risk groups and general population in China between 2013 and 2015.

**Methods:**

This retrospective cohort study conducted an ACF in ten communities of Dongchuan County, located in northeast Yunnan Province between 2013 and 2015; and compared to 136 communities that had passive case finding (PCF). The algorithm for ACF was: 1) screen for TB symptoms among community enrolled residents by home visits, 2) those with positive symptoms along with defined high-risk groups underwent chest X-ray (CXR), followed by sputum microscopy confirmation. TB incidence proportion and the number needed to screen (NNS) to detect one case were calculated to evaluate the ACF strategy compared to PCF, chi-square test was applied to compare the incidence proportion of TB cases’ demography and the characteristics for detected cases under different strategies. Thereafter, the incidence rate ratio (IRR) and multiple Fisher’s exact test were applied to compare the incidence proportion between general population and high-risk groups. Patient and diagnostic delays for ACF and PCF were compared by Wilcoxon rank sum test.

**Results:**

A total of 97 521 enrolled residents were visited with the ACF cumulatively, 12.3% were defined as high-risk groups or had TB symptoms. Sixty-six new TB patients were detected by ACF. There was no significant difference between the cumulative TB incidence proportion for ACF (67.7/100000 population) and the prevalence for PCF (62.6/100000 population) during 2013 to 2015, though the incidence proportion in ACF communities decreased after three rounds active screening, concurrent with the remained stable prevalence in PCF communities. The cumulative NNS were 34, 39 and 29 in HIV/AIDS infected individuals, people with positive TB symptoms and history of previous TB, respectively, compared to 1478 in the general population. The median patient delay under ACF was 1 day (Interquartile range, IQR: 0–27) compared to PCF with 30 days (IQR: 14–61).

**Conclusions:**

This study confirmed that massive ACF was not effective in general population in a moderate TB prevalence setting. The priority should be the definition and targeting of high-risk groups in the community before the screening process is launched. The shorter time interval of ACF between TB symptoms onset and linkage to healthcare service may decrease the risk of TB community transmission. Furthermore, integrated ACF strategy in the National Project of Basic Public Health Service may have long term public health impact.

## Multilingual abstracts

Please see Additional file 1 for translations of the abstract into the five official working languages of the United Nations.

## Background

China has one of the highest tuberculosis (TB) burdens for incident TB cases and multidrug-resistant (MDR)-TB cases in the world [1]. Health organizations made an urgent call to all countries to make an effort to find roughly a third of ‘missing TB cases’ of global prevalence [1, 2].

TB spreads in communities, especially among hard-to-reach populations, the barriers to access diagnosis and receive treatment, in addition to insufficient case identification and reporting, lead to the challenge in the achievement of World Health Organization’s (WHO) End TB Strategy and the United Nations’ (UN) Sustainable Development Goals (SDGs) [3, 4].

Active case finding (ACF) strategy, which entails systematic screening plus clinical evaluation of people with presumptive TB in a target group by using rapid tests or other procedures, plays an important role in TB case finding. A few studies across China showed that ACF increased the number of TB cases four to eight fold compared to passive case finding (PCF) strategy, especially in the elderly [5, 6] and smokers [7]. A systematic review assessed the number of people needed to screen (NNS) in order to detect one case of active TB under ACF strategy, and showed that weighted mean and range of NNS was 669 (15–5594) in the general population, 61 (5–316) for HIV positive individuals, 2223 (range not available) for people with diabetes mellitus (DM), and 40 (7–335) for household contacts in moderate incidence settings [8, 9].

In addition, those detected cases through active screening were more likely to be at an earlier stage of the disease, previous study showed that a higher proportion of patients detected by community survey did not have chest symptoms (cough over 3 weeks) compared with patients detected by health facilities, which of 28% vs 13% in smear-positive TB, and 45% vs 28% in smear-negative TB patients, respectively [10].

The WHO’s operational guide for systematic screening for active TB recommends focusing on high-risk populations rather than indiscriminate mass screening [9].

Our study was conducted in Yunnan in a community-based cohort between 2013 and 2015, to evaluate the utility of ACF compared to PCF in the general community and to estimate the TB incidence proportion, the time from symptom onset to the ACF visit or PCF identification, and from the identification to TB diagnosis. For the ACF strategy, we also estimated the number needed to screen among high-risk populations.

## Methods

### Study design

We conducted this retrospective cohort study in Yunnan Province of China between 2013 and 2015.

### General setting

In 2017, notified TB cases of Yunnan were 27 448 [11]. Dongchuan County, located in northeast Yunnan, is composed of 146 communities and villages; the population was 274 073, 275 362 and 276 993 in years of 2013, 2014 and 2015 respectively. Before this study, in Dongchuan County, 218 TB cases were notified in 2012, giving a prevalence of 79.8/100000, and in the research field of 10 communities, there were 28 TB patients giving a prevalence of 83.8/100000. After the study, without screening intervention the TB prevalence among the 10 communities were 45.4/100000 in 2016 and 35.6/100000 in 2017, and the TB prevalence in PCF communities were 74.9/100000 in 2016 and 63.7/100000 in 2017.

### TB prevalence survey

Our research was part of the field work and applied the information collected from the “Study on TB epidemic and intervention mode in China”, and it was one study of The National Twelfth Five-year Mega-Scientific Projects of Infectious Diseases. Briefly, this TB prevalence survey used multi-stage cluster sampling, and randomly sampled out 10 counties in 10 provinces throughout the nation based on population size and TB prevalence level, Dongchuan County was one of the 10 selected counties [5].

In Dongchuan County, 10 of 146 communities were selected randomly as part of this study for community-based TB screening between 2013 and 2015, other 136 communities performed routine TB detection and diagnosis protocol follow National Tuberculosis Control Program in China (China NTP).

### Active case finding strategy

Registered residents who had resided in the study field for at least 6 months in the past year and unregistered or temporary residents who had lived continuously in the study field for at least 6 months were enrolled for the study. Enrolled residents were excluded if they were unwilling to give consent, loss to follow-up, dead or they had moved out of research field in the secondary and third year of study.

**Fig. 1:**
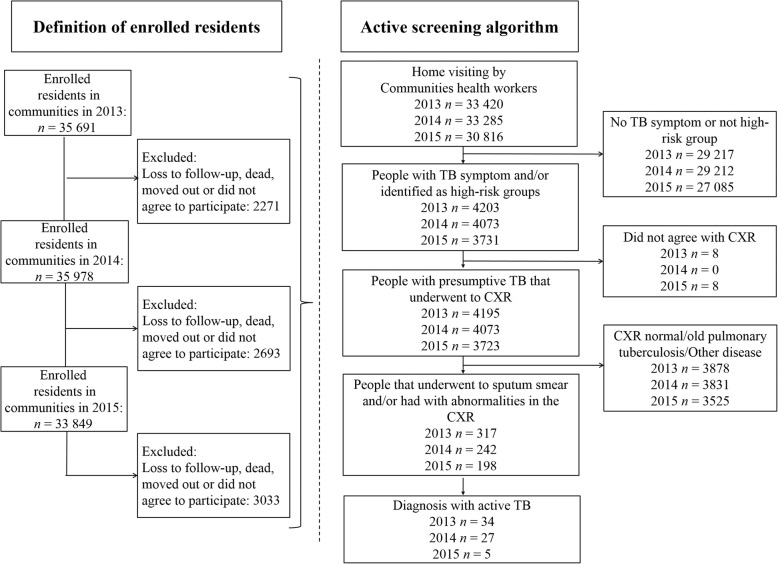
Flow chart of active tuberculosis screening process among communities in Yunnan, 2013–2015 High-risk groups: Elderly, Diabetes mellitus, HIV/AIDS, close contact and history of previous tuberculosis case. CXR: chest X-ray

Home visit was performed by trained community health workers (Fig. 1) who completed the survey to screen for positive TB symptoms (cough or expectoration over 2 weeks, or hemoptysis).

Those with positive TB symptoms or in a high-risk population (elderly age over 65, diabetes mellitus, HIV/AIDS, close contact to an index TB case and history of previous pulmonary or extrapulmonary TB) underwent chest X-ray (CXR). Then, those with TB symptoms or abnormal lung shadows on CXR were requested to submit three sputum samples (one spot sputum, one at night and one on the next morning) for smear tests. After that, those with inconspicuous, indistinguishable CXR shadows or differential diagnosis to other lung diseases, had their CXRs transferred to the national TB diagnosis committee for confirmation after the screening process.

Trained community health workers performed home visit in the secondary and third year of study to identify enrolled residents and screen for positive TB symptoms with identical including and excluding criteria, those with positive TB symptoms or in a high-risk population followed the diagnostic process of the screen in 2013. All diagnosed TB cases were treated and monitored according to China NTP.

### Passive case finding strategy

Aside from the 10 ACF communities, 136 communities in the county performed PCF and were entirely included for this study. Health care workers in Dongchuan County Center of Disease Control and Prevention (CDC) executed passive case finding by checking presumptive TB patients on National Tuberculosis Information Management System (TBIMS), which the people with TB symptoms located in 136 communities outside the 10 ACF communities in Dongchuan County that sought care in health facilities. Presumptive TB patients were traced and transferred to Dongchuan CDC TB clinic for TB diagnosis and treatment. Once TB suspects were referred to or reached the CDC TB clinic, they were diagnosed by local CDC TB clinic doctors according to CXR and sputum test results. All diagnosed TB cases were treated and monitored according to China NTP under PCF setting.

### Data collection and variables

Data under ACF strategy was collected by trained community health workers when they carried out home visits using standardized questionnaires; an online electronic system was designed for data double-entered and to check the consistency. Data were extracted from the primary study previously described.

Variables collected from ACF participants were: sex, age, education level, occupation, ethnicity, body mass index (BMI), TB symptom status (positive, negative), date of TB symptom onset, CXR status (normal, active, inactive, other disease), sputum smear (positive, negative), diagnosis of TB (TB, not TB), category of treatment (new; retreatment); date of medical examination, date of diagnosis, high-risk population category, total population per year in Dongchuan, and total screened population.

TB patients’ information under PCF strategy was extracted from TBIMS and included: sex, age, occupation, ethnicity, date of TB symptom onset, sputum smear, diagnosis of TB, category of treatment, date of the first visit to health care facilities and date of diagnosis.

### Definition of number needed to screen and delays

The number needed to screen (NNS) to detect one case was calculated as the total number of screened people divided by the number of TB cases identified [9].

Patient delay was defined from the onset of TB symptoms to the patient’s first home visit for ACF or to a healthcare facility for PCF. In ACF strategy, people in high-risk groups directly underwent CXR regardless of TB symptoms. Patient delay under ACF without TB symptoms was defined as zero. Diagnostic delay was defined from patient’s first visit to the confirmation of TB diagnosis. Total delay was the sum of patient delay and diagnostic delay [12].

### Data analysis

The demographic description was reported as frequency or proportion for categorical or ordinal variables. Incidence proportion and NNS were calculated per year, for each strategy and for high-risk populations [13]. The chi-square test, continuity correction chi-square test, and Fisher’s exact test were used to test the differences of TB cases’ demography and cumulative incidence under different case finding strategies. Multiple Fisher’s exact test comparisons between high-risk populations were done to test the difference of TB incidence by adjusting *P*-value using the Bonferroni method to control false positive. The incidence rate ratio was calculated to compare high-risk populations with general population. Delays were described by median and interquartile range (IQR) in days; and comparison of delays between different case finding strategies, years and high-risk populations were done with Wilcoxon rank sum test. Differences with *P*-value < 0.05 was considered statistically significant. R software (R Core Team 2016; http://www.R-project.org) was used for statistical analysis. The study complied with the Strengthening the Reporting of Observational Studies in Epidemiology (STROBE) guidelines [14]. Meanwhile, data analysis based on the pragmatic framework for evaluating and measuring the effect of improved case detection strategies of TB [15].

## Results

### Tuberculosis screening results and demographics

There were 92.4% (97 521/105518) of enrolled residents visited by community health workers cumulatively. Among them, 12.3% (12 007/97521) were in a high-risk group or had TB symptoms (Fig. 1). There were 66 new TB cases identified under ACF strategy.

The cumulative TB incidence among all people screened in ACF was 67.7/100000 and the cumulative NNS was 1478. Demographic characteristics of the screened population are shown in Table 1.

**Table 1:** Demographic characteristics of enrolled residents and tuberculosis cases diagnosed by active case finding screening in Yunnan, 2013–2015

Characteristic	Cumulative enrolled resident(*n*)	Cumulative tuberculosis diagnosis (*n*)	Cumulative tuberculosis incidence ^*a*^	*χ* ^*2*^	*P* value	NNS ^*b*^
Sex						
male	47 515	38	79.9	2.1	0.15	1250
female	50 006	28	55.9			1786
Age (years)						
< 15	19 069	1	5.2	183.0	< 0.01	19 069
15–34	21 288	6	28.2			3548
35–64	46 147	17	36.8			2715
≥ 65	11 017	42	381.2			262
Occupation						
children/students/teachers	28 534	5	17.5		< 0.01^ǁ^	5707
farmer/worker	17 897	6	33.5			2983
medical staff	871	0	0.0			
government employees	2972	0	0.0			
retired	13 534	33	243.8			410
unemployed	26 886	20	74.4			1344
others	6827	2	29.3			3414
Education						
no schooling	9031	17	188.2		< 0.01^ǁ^	531
primary school	23 394	18	76.9			1300
middle school	36 035	20	55.5			1802
high school	16 170	5	30.9			3234
college and above	12 065	6	49.7			2011
missing	826	0	0.0			
Ethnicity						
Han	89 577	58	64.8	1.39	0.24	1544
Other minority	7944	8	100.7			993
BMI (kg/m^2^)^c^						
< 18.5	5573	11	197.4		< 0.01^ǁ^	507
18.5–24.9	52 990	47	88.7			1128
25.0–29.9	13 720	5	36.4			2744
≥ 30.0	1435	1	69.7			1435
missing	23 803	2	8.4			11 902

a: TB Incidence proportion = new TB cases/population in ACF area× 100 000

b: NNS = number needed to screen to detect one case

c: BMI = body mass index

ǁ Fisher’s exact test

Demographic characteristics of TB cases identified by ACF and PCF are shown in Table 2. Among 522 TB patients detected in the county, 18.1% (12/66) were smear positive and 15.1% (10/66) were retreatment pulmonary TB under ACF; compared to 28.5% (130/456) smear positive (*P* = 0.08); and 3.1% (14/456) retreatment pulmonary TB (*P* <  0.01) under PCF strategy.

**Table 2:** Characteristics of tuberculosis cases identified by active and passive case finding strategies in Yunnan, 2013–2015

Characteristic	ACF*		PCF*		*χ* ^*2*^	*P* value
	Number of cases (*n*)	Proportion (%)	Number of cases (*n*)	Proportion (%)		
All	66		456			
Sex						
male	38	57.6	305	66.9	2.2	0.14
female	28	42.4	151	33.1		
Age (years)						
< 15	1	1.5	6	1.3		< 0.01^ǁ^
15–34	6	9.1	110	24.1		
35–64	17	25.8	246	54.0		
≥ 65	42	63.6	94	20.6		
Occupation						
children/students/teachers	5	7.6	19	4.2		< 0.01^ǁ^
farmer/worker	6	9.1	402	88.2		
medical staff	0	0.0	2	0.4		
government employees	0	0.0	5	1.1		
retired	33	50.0	21	4.6		
unemployed	20	30.3	0	0.0		
others	2	3.0	7	1.5		
Ethnicity						
Han	58	87.9	434	95.2	4.4	0.04^ǂ^
Other Minority	8	12.1	22	4.8		
Sputum smear						
negative	54	81.8	326	71.5	3.1	0.08
positive	12	18.2	130	28.5		
Category of treatment						
new case	56	84.9	442	96.9	16.5	< 0.01^ǂ^
retreatment	10	15.1	14	3.1		

*ACF = active case finding, PCF = passive case finding

ǁ Fisher’s exact test

ǂ Continuity correction

### Case detection under ACF and PCF

The TB incidence proportion in ACF communities decreased between 2013 and 2015 but was stable over time in PCF communities (Table 3). Meanwhile, the difference of NNS between PCF and ACF strategies was 700, 370 and 4643 in the year of 2013, 2014 and 2015, respectively. The difference of cumulative incidence and prevalence between ACF (67.7/100000) and PCF (62.6/100000) was not significant (*P* = 0.54).

**Table 3:** Number needed to screen and tuberculosis incidence proportion or tuberculosis prevalence rate for active and passive case finding strategies in Yunnan, 2013–2015

Year	Screen strategy	Screened enrolled residents under ACF/ Population under PCF, *n*	TB diagnosed, *n*	TB incidence proportion/ prevalence ^*a*^	Rate ratio ^*b*^	95% *CI* of *RR*^*c*^	NNS ^*d*^
2013	ACF*	33 420	34	101.7	1.7	1.2–2.5	983
	PCF*	240 653	143	59.4	ref		1683
2014	ACF	33 285	27	81.1	1.3	0.8–1.9	1233
	PCF	242 077	151	62.4	ref		1603
2015	ACF	30 816	5	16.2	0.2	0.08–0.6	6163
	PCF	246 177	162	65.8	ref		1520

*ACF = active case finding, PCF = passive case finding; TB = tuberculosis

a: TB Incidence proportion = new TB cases/screened population in ACF area×100 000; TB prevalence = TB cases/population in PCF area×100 000

b: Rate ratio = incidence proportion under ACF/ prevalence under PCF

c: *CI* = confidence interval; *RR* = rate ratio

d: NNS = number needed to screen to detect one case

### TB screening results in high-risk groups

**Fig. 2:**
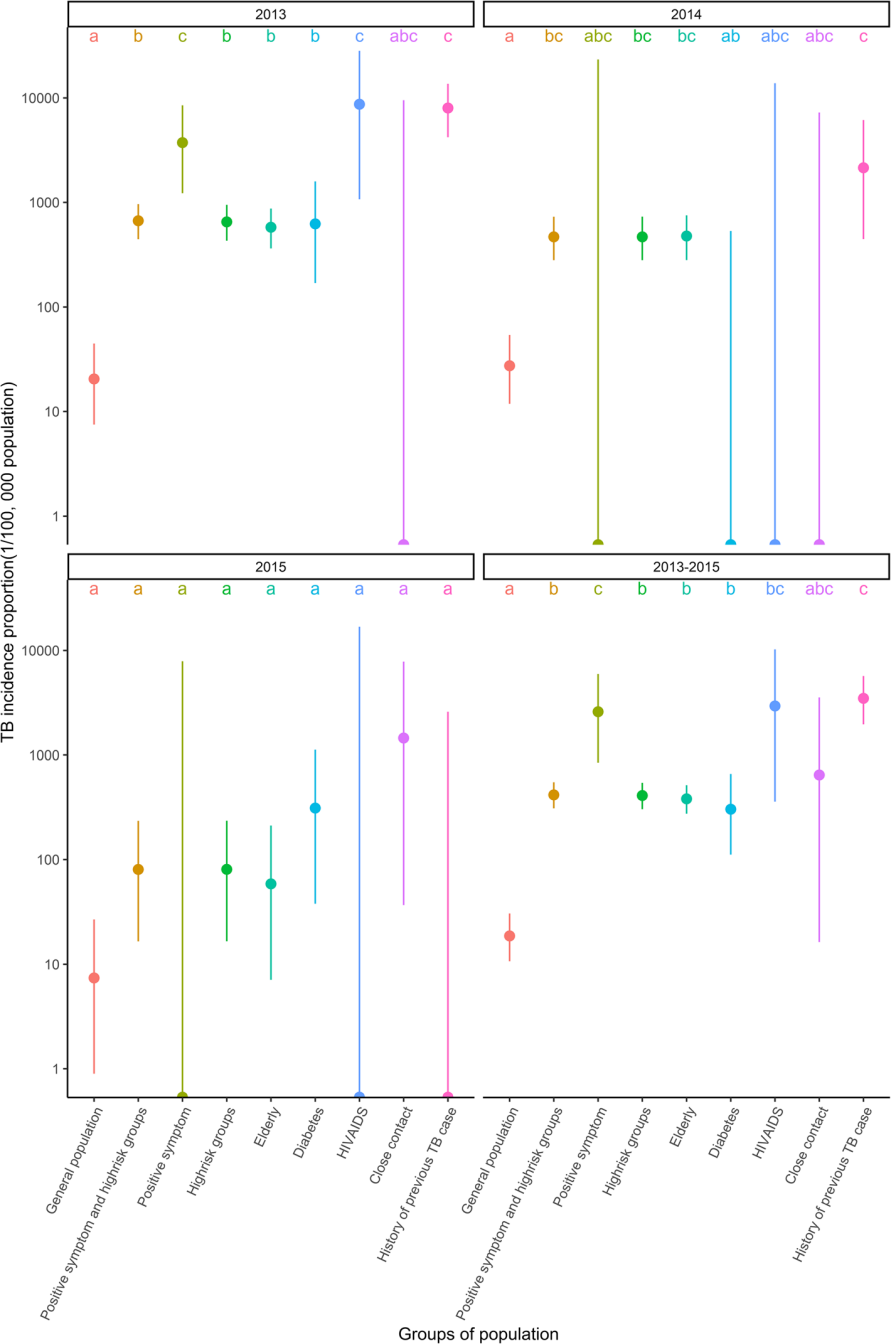
Tuberculosis incidence proportion, 95% confidence intervals and pairwise comparison of new tuberculosis cases in high-risk groups of active case finding strategy in Yunnan, 2013–2015 High-risk groups: Elderly, Diabetes mellitus, HIV/AIDS, close contact and history of previous tuberculosis case. Pairwise *χ*^2^ tests results were summarized as compact letter display, different letters represented statistically significant difference between groups. *Log transformed with Y-axis

**Fig. 3:**
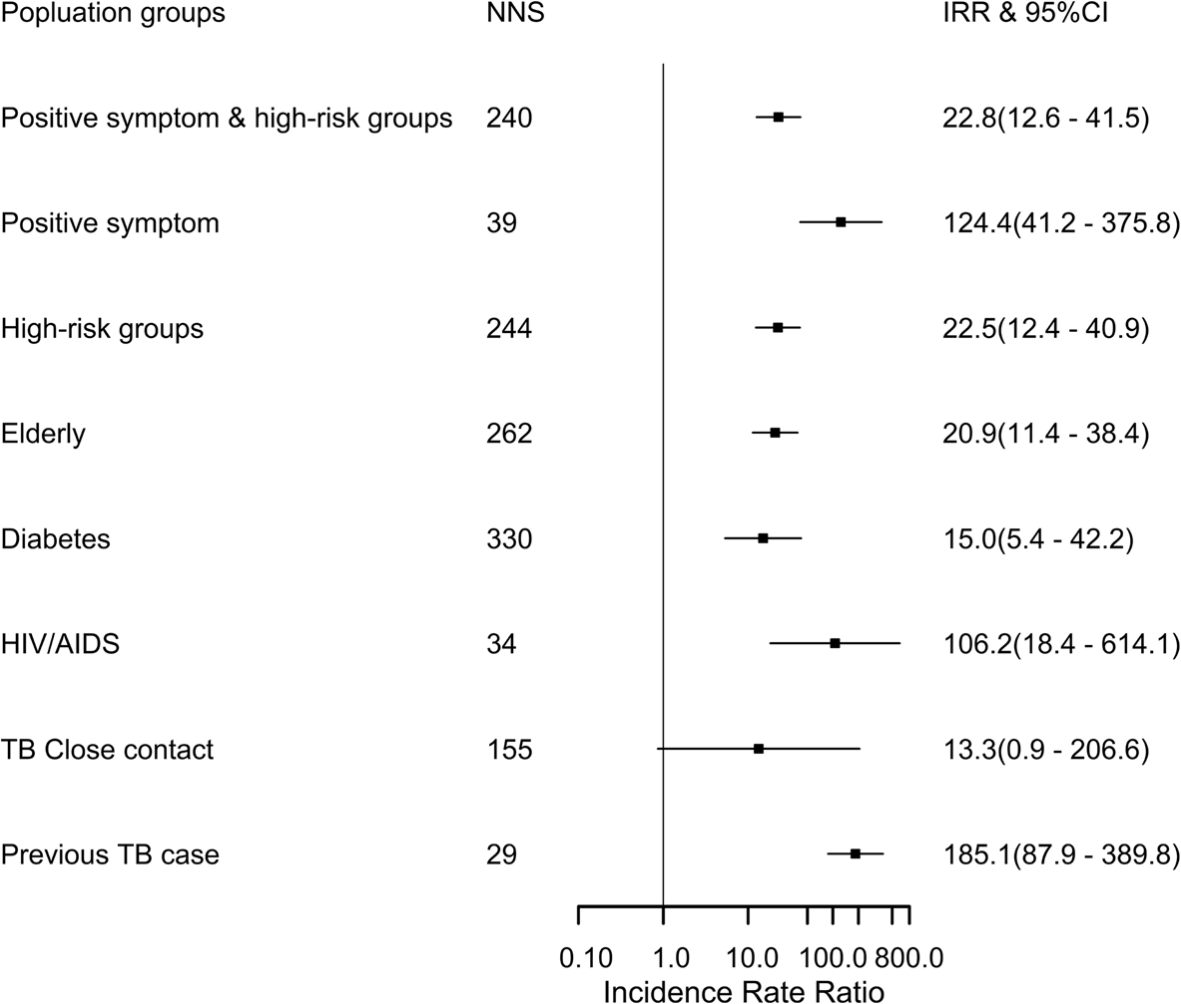
Number needed to screen, incidence rate ratios and 95% confidence intervals for high-risk populations in Yunnan, 2013–2015 NNS: Number needed to screen to detect one tuberculosis case. IRRs: Incidence rate ratio of high-risk population compared to general population in active case finding strategy; *CI*: confidence intervals. High-risk groups: Elderly, Diabetes mellitus, HIV/AIDS, close contact and history of previous tuberculosis case. *Log transformed with X-axis

Cumulative TB incidence were 18.7/100000 in general population and 416.4/100000 in people with symptoms or in a high-risk population (Fig. 2). However, the highest TB incidence proportion was in HIV/AIDS infected individuals, people with positive TB symptoms and history of previous TB (Additional file 2). The incidence rate ratios showed that the incidence proportion was 100 times higher in these groups than the incidence proportion in general population (Fig. 3).

### Patient and diagnostic delay

**Fig. 4:**
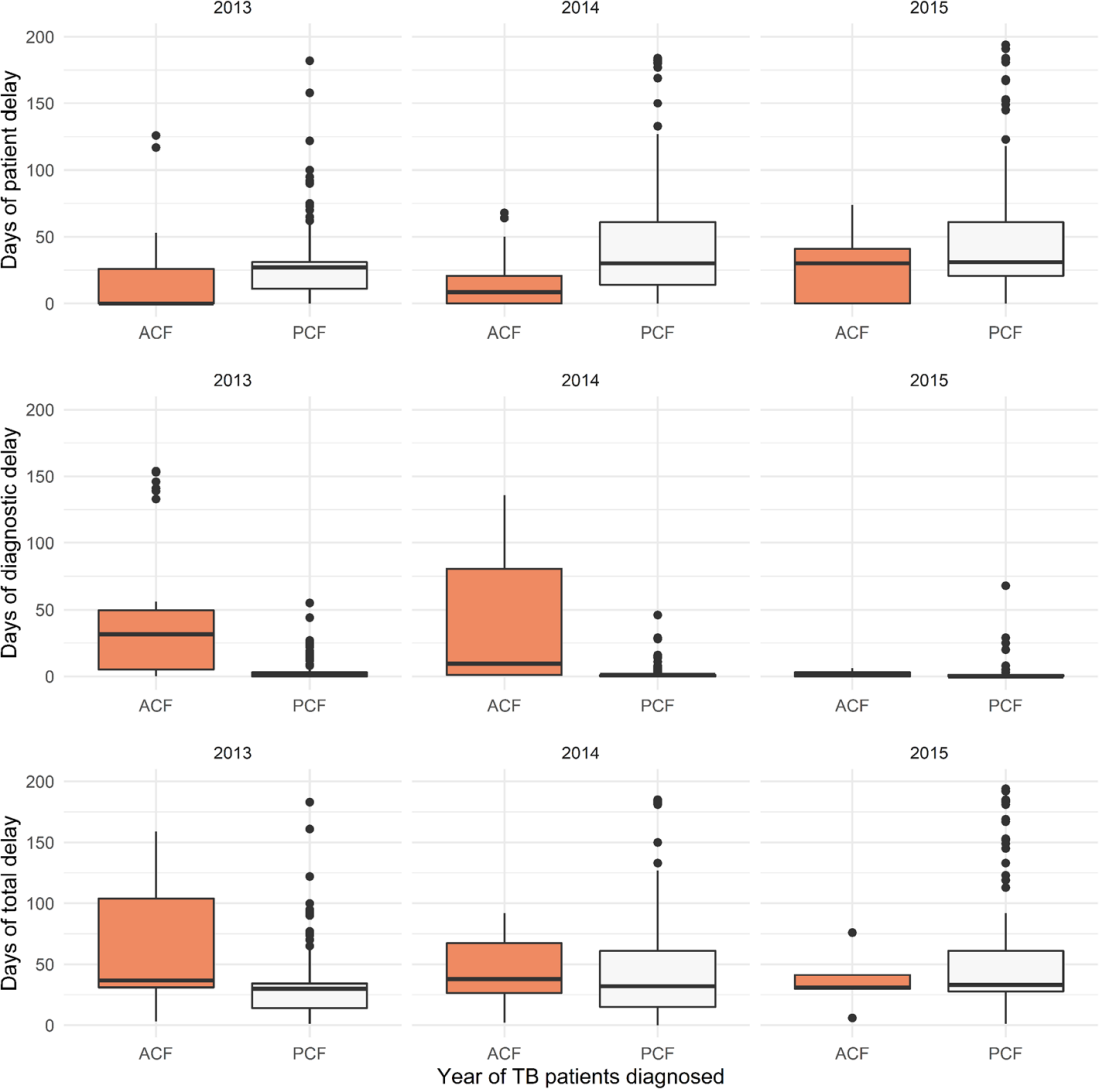
The patient, diagnostic and total delays stratified by case finding strategies and the year of tuberculosis diagnosis in Yunnan, 2013–2015 Days of patient delay: Date from the onset of tuberculosis symptoms to date of the patient’s first home visit for ACF or date to a healthcare facility for PCF. Days of diagnostic delay: Date of patient’s first visit to date of the confirmation of tuberculosis diagnosis by sputum smear or culture. Days of total delay: The sum of patient delay and diagnostic delay. * Wilcoxon rank sum test showed *P*-value < 0.05 between different case finding strategies between 2013 and 2015

The patient delay in ACF was 1 day (IQR: 0–28) and in PCF was 30 days (IQR: 14–61) (*P* <  0.01). Meanwhile, median diagnostic delay for ACF was 22 days (IQR: 3–98) and for PCF was 1 day (IQR: 0–1) (*P* <  0.01). Overall, the median total delay was 39 days (IQR: 31–92) for ACF and 31 days (IQR: 17–61) for PCF (*P* <  0.01) (Fig. 4).

## Discussion

This study conducted a continuous longitudinal TB screening among residents, which in 3 yrs evaluated more than 90 000 residents for active case finding in China. We found that ACF is useful for high-risk groups, with lower numbers needed to screen to detect a TB case compared to PCF. Furthermore, the patient delay was shortened under ACF strategy.

Our study showed that continuous active screening strategy detected different numbers of TB cases among screened communities, yet had an impact on local TB epidemic status. Although there was no significant difference for the cumulative TB incidence or prevalence between ACF and PCF area during the screening time frame, the decrease for the number of cases detected in 2015 could be explained by earlier detection of most TB cases in 2013 and 2014 caused by the massive screening effort. Moreover, between 2012 and 2017, the notification rate of TB decreased by 57.5% (from 83.8/100000 to 35.6/100000) in ACF and 19.6% (from 79.2/100000 to 63.7/100000) in PCF areas. Enhanced continuous case finding strategy and health education about TB for screened people during the ACF, combined with the decreased patient delay resulted in reducing the risk of TB transmission among communities. Therefore, our findings indicate that over time the ACF approach would contribute to decrease the TB incidence. This might imply TB was on the path of elimination in these 10 communities in Dongchuan due to full coverage ACF. The DETECTB study found similar results to us – after six rounds of ACF, the TB burden in the community had fallen by more than 40% compared to rates before intervention [16].

The selection of cough, expectoration over 2 weeks, or hemoptysis as inclusion criteria for CXR was following China NTP [17], which showed 43.2 and 98.6% of sensitivity and specificity in all screened participants and a better performance in age over 65 years, this primary screening test was rapid, convenient and resource-saving for massive population screening [18]. In our study, symptomatic suspects yield 7.5% (5/66) to all screened out TB patients.

The proportion of 18.1% smear-positive cases was comparable with the national TB prevalence surveys in 2010, which was 14.8% (188/1310) among active pulmonary TB detected [19], but lower than 32.0% of bacteriologically confirmed cases among TB cases under PCF routinely reported [1]. Beyond that, by keeping stable screening personnel and teams, we maintained high sensitivity of CXR to reduce missed cases. We controlled misdiagnosis of negative smear cases by transferring these CXRs to national TB diagnosis committee for confirmation, still, there was a drop-off in TB-confirmed cases among persons with abnormal CXR in the final year of the intervention, 2.5% in 2015 compared to 10.7 and 11.5% in 2013 and 2014. This study also found 16 cases in the general population with no symptoms and no high-risk factors, which makes up 24.2% (16/66) of the cases found. There was a high chance that without ACF of the general population, a sizable share of these 16 asymptomatic persons would have been missed.

Based on our results, the community ACF was useful for high-risk groups as the IRR was higher and the NNS was lower compared to the general population. Globally, different studies have shown that the ACF strategy increases tuberculosis case detection in high-risk groups, especially in HIV positive individuals and people with DM [20–23]. The explanation for ACF being more effective in high-risk groups is that the prevalence and incidence proportion are higher than in general population. Our study showed that cumulative incidence of TB in HIV/AIDS was 2941/100000 population, similar to the high incidence reported in countries with high burden of TB and HIV, 0.8/100 per person-years in Tanzania, 1839/100000 to 1936/100000 population in Kenya [24, 25], and 3.3 to 7.4% in newly diagnosed with HIV and known HIV positive individuals in South Africa [26]. The high prevalence and burden of both TB and HIV/AIDS in Yunnan Province implies that optimized ACF algorithm might improve TB detection among people with HIV/AIDS and contribute to the TB elimination goal [27, 28].

Among people with DM, TB incidence proportion was 622/100000 population in the first year of screening and the cumulative incidence in the 3 yrs of ACF was 303/100000 in our research, similar to the prevalence of 389/100000 in Taiwan, but lower than the overall TB prevalence in Asian studies [29, 30]. In addition, our cumulative incidence was 2.9-fold higher than a local study that reported 102/100000 among individuals with DM [31], which the baseline TB prevalence in our study was 2.2-fold higher than a local study of 36/100000 population regardless of different socioeconomic factors. The IRR of TB among people with DM was higher than the general population and it shows that DM increases the risk of TB, as it has been reported by systematic reviews, regardless of study design and population [32, 33].

ACF was most valuable for symptomatic and high-risk groups in the first year of screening, during which TB incidence proportion in these groups was 33-fold higher than the general population; whereas the incidence proportion was 17 and 11 times higher than general population in the second and third year. The increase of NNS in symptomatic and high-risk groups over time indicates that ACF contributed to decreasing TB burden in these groups.

In addition, we found that ACF reduced the time interval between TB symptom onset and the visit by active screening algorithm compared to PCF. However, the diagnostic delay under ACF strategy was caused mainly by the time consumed by transferring the patients’ CXR to the national TB diagnosis committee to confirm the diagnosis. Therefore, ACF diagnostic delay could be shortened by decentralization of the diagnosis, and training healthcare workers at the county level to simplify the process of diagnosis confirmation. Another useful strategy to decrease the time for diagnostic delay is the ACF using mobile chest radiography, which has improved screening coverage for tuberculosis identification and reduced delay among hard-to-reach populations, but the effect of increased case detection in the long term is still unclear [34, 35]. Finally, the use of machine-learning algorithms for CXR classification it has shown to be a useful tool to classify TB [36].

Regarding PCF, the patient’s delay for the onset of TB symptom to seek healthcare in the facility is still a challenge for TB diagnosis in hard-to-reach community populations. A meta-analysis showed that living in rural areas was a risk factor for both patient and diagnostic delay under PCF routine work [12, 37]. Local research also showed that using PCF, the median delay to reach a directly observed treatment short-course strategy (DOTS) facility was 57 days, and TB confirmation was 2 days in Yunnan between 2008 and 2013. This is important because the delay to treatment initiation has a strong relationship with intra-household TB transmission [38, 39].

In addition, the proportion of recurrent TB cases was substantially higher in ACF than in PCF. The TB suspected patients were more willing to seek health care when TB symptoms first appeared under PCF settings [40]. Nonetheless, ACF reached the hard-to-reach populations who preferred waiting passively for health care given that they previously had unfavorable TB treatment outcome. The study did not carry out drug susceptibility test (DST), recurrence or relapse TB cases may have been Rifampicin Resistance/MDR-TB, hence ACF strategy should include DST, in particular for high-risk groups, taking into account that China has one of the highest RR/MDR-TB rates worldwide.

Our study has the strength that this is a community-based population study with a relatively stable cohort (over 30 000 population per year), and it was strictly designed and operated by a stable screening personnel team. Although the number of screened enrolled residents were discrepant over 3 yrs, the exclusion of enrolled residents who moved out or were unwilling to participate in the intervention did not change the demographics of the screened population. The male to female ratio maintained at 1 to 1.05 and the proportion of age over 65 was stable at 11.4, 11.4 and 11.1% between years of 2013 and 2015, which made screening cohort comparable over time.

However, the study has the limitation that among 66 patients diagnosed in ACF strategy, only 18.1% had confirmation by sputum smear, therefore new rapid diagnostic tools like GeneXpert MTB/RIF should be introduced to ACF, as they have demonstrated feasibility and effectiveness as an additional diagnostic tool to ACF [6]. Particularly, in high-risk groups including HIV/AIDS and previous TB cases, new tools like GeneXpert MTB/RIF with high sensitivity and specificity would increase yield and optimize ACF algorithm by combining with CXR and sputum test, it would be more resource intensive but worth it. We could not compare TB disease risk factors associated with ACF and PCF strategy because the data for diabetes status, BMI and close contact status under PCF were not accessible in TBIMS. Thirdly, although the screening cohort remained stable in the study time frame, potential demographic confounders like income, tobacco use, and drinking history were not addressed in the study. Fourthly, demographic subgroups with potentially high TB incidence, such as low BMI and minority groups were not introducing CXRs for diagnosis, which might lead to underestimated incidence for these subgroups. Further study should address this.

Yunnan province carried out The National Project of Basic Public Health Service (BPHS). BPHS covers elderly and diabetes mellitus among community populations, and requests that they undergo physical examination along with an annual visit by a community healthcare worker. The findings of our study show that the elderly and people with DM would benefit from ACF TB detection strategy. Therefore, BPHS may integrate the ACF strategy, including TB symptom screening and CXRs, when elderly and DM patients undergo routine physical examination according to available resources.

## Conclusions

Our study found that community-based active case finding is a useful strategy to detect tuberculosis in high-risk groups like elderly, or people with HIV/AIDS, diabetes mellitus or a history of prior TB, as the number needed to screen was lower and the incidence rate ratio was higher compared to the general population in a moderate TB prevalence setting. As WHO recommends, indiscriminate ACF is not recommended for general population, but massive screening contributed to a sizeable detection of missing cases that were without symptoms or high-risk factors. Additionally, ACF strategy significantly reduced the time between TB symptom onset and access to healthcare service, however, time from home visit to TB diagnosis needs to improve.

## Supplementary information

**Additional file 1.** Multilingual abstracts in the five official working languages of the United Nations.

**Additional file 2: Table S1.** Incidence proportion of new TB cases and number needed to screen in high-risk groups of active case finding strategy in Yunnan, 2013–2015.

## Abbreviations

ACF: Active case finding
AIDS: Acquired immune deficiency syndrome
BMI: Body mass index
BPHS: National Project of Basic Public Health Service
CDC: Center of Disease Control and Prevention
China NTP: National Tuberculosis Control Program in China
CXR: Chest X-ray
DOTS: Directly observed treatment short course strategy
DST: Drug susceptibility test
HIV: Human immuno-deficiency virus
IQR: Interquartile range
IRR: Incidence rate ratio
MDR-TB: Multidrug resistant tuberculosis
NNS: Number needed to screen to detect one case
PCF: Passive case finding
SDGs: Sustainable Development Goals
TB: Tuberculosis
TBIMS: National Tuberculosis Information Management System
UN: United Nations
WHO: World Health Organization
