# Correction to: Role of community-based active case finding in screening tuberculosis in Yunnan province of China

**DOI:** 10.1186/s40249-020-0625-6

**Published:** 2020-01-18

**Authors:** Jin-Ou Chen, Yu-Bing Qiu, Zulma Vanessa Rueda, Jing-Long Hou, Kun-Yun Lu, Liu-Ping Chen, Wei-Wei Su, Li Huang, Fei Zhao, Tao Li, Lin Xu

**Affiliations:** 1 Division of tuberculosis control and prevention, Yunnan Center for Disease Control and Prevention, Kunming, China; 2 Universidad Pontificia Bolivariana, Medellín, Colombia; 3 Clinical trail and research center of Beijing hospital, Beijing, China; 4 Chinese Center for Disease Control and Prevention, Beijing, China

**Correction to: Infect Dis Poverty**

**https://doi.org/10.1186/s40249-019-0602-0**

In the original publication of this article [1] we noticed the Fig. 4 was incorrect. The correct Fig. 4 is as below:

**Fig. 4:**
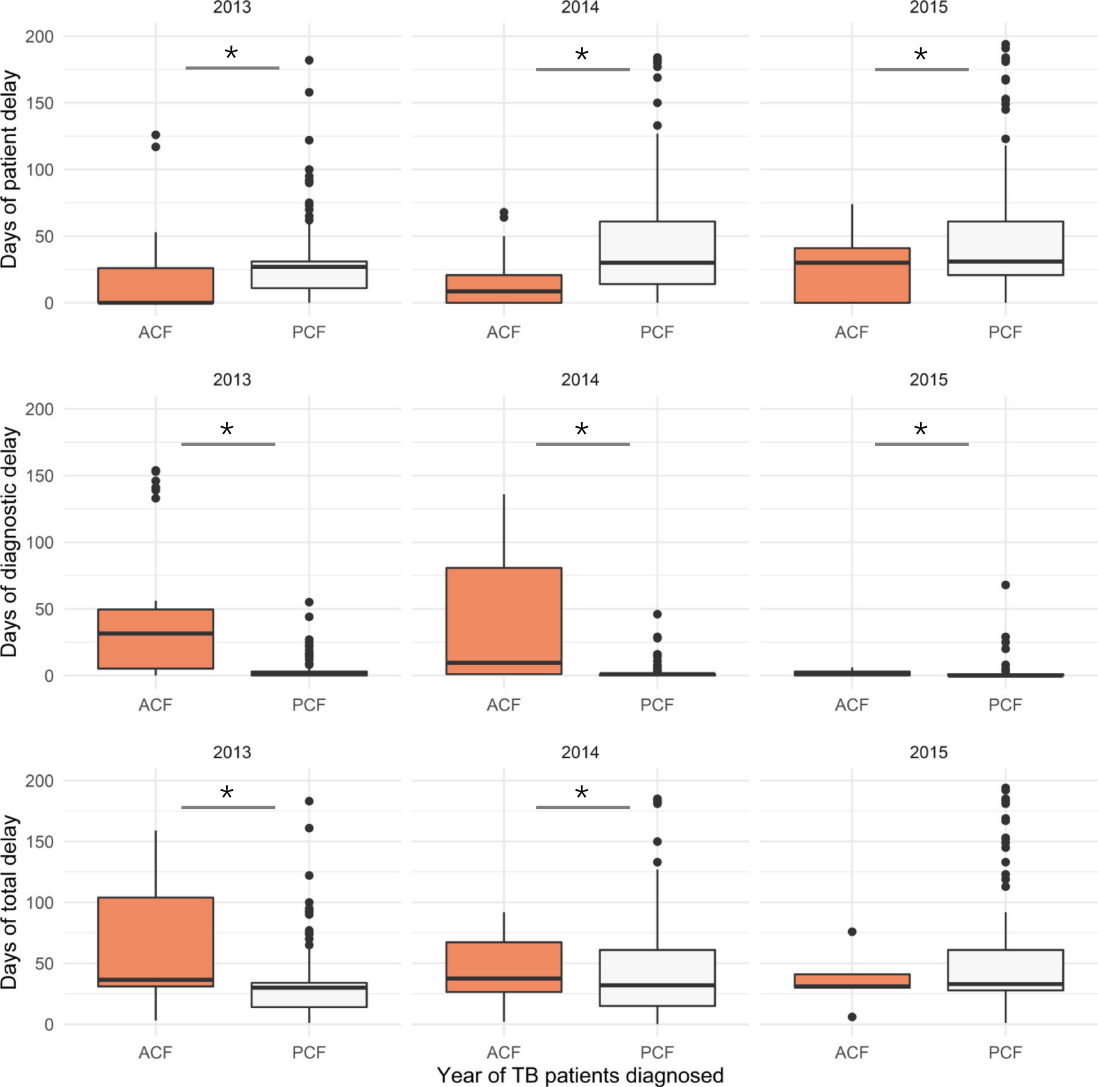
The patient, diagnostic and total delays stratified by case finding strategies and the year of tuberculosis diagnosis in Yunnan, 2013–2015 Days of patient delay: Date from the onset of tuberculosis symptoms to date of the patient’s first home visit for ACF or date to a healthcare facility for PCF. Days of diagnostic delay: Date of patient’s first visit to date of the confirmation of tuberculosis diagnosis by sputum smear or culture. Days of total delay: The sum of patient delay and diagnostic delay. * Wilcoxon rank sum test showed *P*-value < 0.05 between different case finding strategies between 2013 and 2015
