# Supplementary material for: Role of community-based active case finding in screening tuberculosis in Yunnan province of China
Source: Infect Dis Poverty. 2019 Oct 29;8:92. doi: 10.1186/s40249-019-0602-0 (PMC6819334; doi:10.1186/s40249-019-0602-0)
Supplement: Supplementary file 1 — Additional file 1. Multilingual abstracts in the five official working languages of the United Nations. [file 40249_2019_602_MOESM1_ESM.pdf]

Translation of the abstract into the five official working languages of the United Nations

دور الكشف النشط للحالات القائم على المجتمع في فحص السل في مقاطعة يونان الصينية

جين-أوو تشن، ويو-بن كيو، وزولما فانيسا رويدا، وجينغ-هوى طويل، وكون-يون لو، وليو-بينغ تشن، ووي-وي سو، ولي هوانغ، وفاي تشاو، وتاو لي، ولين شو

الملخص

خلفية: تؤدي العوائق التي تحول دون الوصول إلى التشخيص وتلقي العلاج، بالإضافة إلى عدم كفاية تحديد الحالات والإبلاغ عنها، إلى انتشار السل في المجتمعات، وخاصة بين السكان الذين يصعب الوصول إليهم. قيمت هذه الدراسة استراتيجية الكشف النشط للحالات القائم على المجتمع للكشف عن حالات السل بين الفئات المعرضة للخطر والسكان عامة في الصين بين عامي 2013 و 2015.

الطرق: أجريت دراسة المجموعة هذه بأثر رجعي الكشف النشط للحالات في عشر مجتمعات في مقاطعة دونغتشوان، والتي تقع في شمال شرق مقاطعة يونان بين عامي 2013 و 2015 ؛ وبالمقارنة مع 136 مجتمعًا كان فيه الكشف غير النشط للحالات. كانت خوارزمية الاكتشاف النشط للحالات: 1) فحص أعراض السل بين السكان المسجلين في المجتمع من خلال الزيارات المنزلية، و 2) خضع أولئك الذين يعانون من أعراض وضعية جنبًا إلى جنب مع الفئات المعرضة للخطر المحددة لفحص الصدر بالأشعة السينية (CXR) وتلاها تأكيد فحص بلغم مجهري. تم حساب نسبة الإصابة بالسل والعدد اللازم للفحص للكشف عن حالة واحدة لتقييم استراتيجية الكشف النشط للحالات مقارنة مع الكشف غير النشط للحالات، وتم تطبيق اختبار مربع تشي لمقارنة ديموغرافيا نسبة حدوث حالات السل وخصائص الحالات المكتشفة تحت استراتيجيات مختلفة. بعد ذلك، تم تطبيق نسبة معدل الإصابة وعدة اختبارات فيشر دقيقة لمقارنة نسبة الإصابة بين عامة السكان والمجموعات المعرضة للخطر. تمت مقارنة تأخيرات المرضى والتشخيص للكشف النشط لحالات والكشف غير النشط للحالات مع اختبار مجموع رتبة ويلكسون.

النتائج: تمت زيارة ما مجموعه 521 97 من السكان المسجلين مع الاكتشاف النشط للحالات بشكل تراكمي، وتم تحديد 12.3 ٪ على أنها مجموعات عالية الخطورة أو لديهم أعراض سل. تم الكشف عن ستة وستين من مرضى السل الجدد باستخدام الكشف النشط للحالات. لم يكن هناك فرق كبير بين نسبة الإصابة بالسل التراكمي للكشف النشط للحالات (67.7 / 100000 من السكان) وانتشار الكشف النشط الحالات (62.6 / 100000 نسمة) خلال 2013 إلى 2015، على الرغم من انخفاض نسبة الإصابة في مجتمعات الكشف النشط للحالات بعد ثلاث جولات من الفحص النشط، بالتزامن مع الانتشار الذي بقي ثابتًا في مجتمعات الكشف غير النشط للحالات. كان العدد اللازم للفحص التراكمي هو 34 و 39 و 29 في الأفراد المصابين بفيروس نقص المناعة البشرية / الإيدز، والأشخاص الذين يعانون من أعراض السل الوضعية والأشخاص الذين لديهم تاريخ إصابة سل سابق، على التوالي، مقارنة مع 1478 في عموم السكان. كان التأخير المتوسط للمريض بموجب الكشف النشط للحالات يومًا واحدًا (مدى ما بين الربيعات: 0-27) مقارنةً بالكشف غير النشط مع مدة 30 يومًا (مدى ما بين الربيعات: 14–61).

الاستنتاجات: أكدت هذه الدراسة أن الكشف النشط عن الحالات الضخم لم يكن فعالًا في عموم السكان في حالات انتشار السل المعتدل. يجب أن تكون الأولوية هي تعريف واستهداف الفئات عالية الخطورة في المجتمع قبل بدء عملية الفحص. قد يقلل الفاصل الزمني الأقصر للكشف النشط عن الحالات بين ظهور أعراض السل والربط بخدمة الرعاية الصحية من خطر انتقال مرض السل إلى المجتمع. علاوة على ذلك، قد يكون لاستراتيجية الكشف النشط عن الحالات المتكاملة في المشروع الوطني لخدمة الصحة العامة الأساسية تأثير طويل المدى على الصحة العامة.

Translated from English version into Arabic by Amira Samai, revised by Muhannad Al-Bayk, through

TRANSLATORS
WITHOUT BORDERS

中国云南省基于社区的患者主动发现在结核病筛查中的作用

陈金瓯，邱玉冰，祖玛.凡妮莎.鲁达，侯景龙，卢昆云，陈留萍，苏玮玮，黄莉，赵飞，李涛，许琳

摘要

引言：诊断可及性差和接受治疗的障碍，以及病例发现和报告的不足，导致结核病在社区内，特别是在难以接触

的人群中持续传播。本研究于 2013-2015 年应用结核病主动发现策略进行社区筛查，并对该策略下中国社区内高危人群和一般人群的筛查效果进行评估和比较。
方法：本研究采用回顾性队列研究的方法，于 2013-2015 年，在云南省东北部的东川市十个社区连续开展结核病社区主动筛查，并与该市 136 个应用被动发现患者策略的社区进行比较。主动发现策略的流程是：1）通过家访对纳入研究的社区居民进行结核病症状筛查；2）对筛出的结核病可疑症状者和高危人群开展胸部 X 光检查，随后进行痰涂片镜检确诊。用发病比例和发现一例患者所需筛查例数评估结核病主动和被动发现策略，卡方检验用于比较主动发现策略下的人口学特征差异和两种策略发现的患者特征差异；其后，应用发病率比和校正多重确切概率检验比较主动发现下一般人群和高危人群的发病差异。利用秩和检验比较不同策略下患者延迟和诊断延迟的差别。
结果：主动筛查共累计家访 97 521 名社区居民，其中 12.3%为高危人群或结核病可疑症状者。主动筛查共发现 66 例结核病患者。2013-2015 年，虽然开展三轮主动筛查的社区结核病发病比例有所下降，同期被动发现社区发病比例保持稳定；但主动发现社区的累计发病比例(67.7/100 000)与被动发现社区(62.6/100 000)相比无统计学差异。筛查 34，39 和 29 人即可分别在 HIV/AIDS 患者、结核病可疑症状者和既往结核病患者中发现一例患者，而在一般人群中，需要筛查 1478 人才能发现一例患者。在主动发现策略下，患者出现症状到就诊的延迟中位数为 1 天（四分位数间距：0～27 天）；在被动发现策略下此延迟为 30 天（四分位数间距：14～61 天）。
结论：本研究确证了在中等结核病负担情况下，对社区一般人群开展大规模主动筛查是低效的。当务之急是在开展筛查前明确社区中的结核病高危人群组。在主动发现策略下患者出现症状到就诊的时间缩短有助于减低结核病在社区内传播的风险。此外，将结核病主动发现策略与基本公共卫生服务整合将会具有长期的公共卫生意义。

Translated from English version into Chinese by Jin-Ou Chen.

Rôle de la recherche communautaire dans le dépistage de cas actifs de tuberculose dans la province du Yunnan en Chine

Jin-Ou Chen, Yu-Bing Qiu, Zulma Vanessa Rueda, Jing-Long Hou, Kun-Yun Lu, Liu-Ping Chen, Wei-Wei Su, Li Huang, Fei Zhao, Tao Li, Lin Xu

Résumé

Contexte: Les obstacles pour accéder au diagnostic et recevoir un traitement, en plus de l'insuffisance de l'identification et de la déclaration des cas, entraînent la propagation de la tuberculose (TB) dans les communautés, surtout parmi les populations difficiles à atteindre. Cette étude a évalué une stratégie communautaire de dépistage actif des cas de tuberculose chez les groupes à haut risque et la population chinoise en général, entre 2013 et 2015.
Méthodologie: Cette étude de cohorte rétrospective a mené une recherche communautaire de cas actifs dans dix communautés du comté de Dongchuan, situées dans le nord-est de la province du Yunnan entre 2013 et 2015 ; et comparé à 136 communautés qui avaient une recherche passive de cas (PCF). L'algorithme de l'ACF était le suivant : 1) dépistage des symptômes de la tuberculose chez les résidents inscrits dans la communauté par des visites à domicile ; 2) ceux qui présentaient des symptômes positifs ainsi que les groupes à risque élevé définis ont subi une radiographie pulmonaire (RXC), suivie d'une confirmation par microscopie expectorale. La proportion de l'incidence de la tuberculose et le nombre nécessaire pour le dépistage (NNS) pour détecter un cas ont été calculés pour évaluer la stratégie ACF par rapport au PCF, le test du chi carré a été appliqué pour comparer la proportion d'incidence de la démographie des cas de tuberculose et les caractéristiques des cas détectés selon différentes stratégies. Par la suite, le ratio du taux d'incidence (TRI) et plusieurs tests exacts de Fisher ont été appliqués pour comparer la proportion d'incidence entre la population générale et les groupes à risque élevé. Les retards des patients et les retards de diagnostic pour l'ACF et le PCF ont été comparés par le test de Wilcoxon à la moyenne de classement.

Résultats: Au total, 97 521 résidents inscrits ont reçu des visites cumulatives au FAC, dont 12,3 % ont été définis comme des groupes à risque élevé ou présentant des symptômes de la tuberculose. Soixante-six nouveaux cas de tuberculose ont été détectés par ACF. Il n'y avait pas de différence significative entre la proportion d'incidence cumulative de la tuberculose pour le FAC (67,7/100 000 habitants) et la prévalence du FPC (62,6/100 000 habitants) entre 2013 et 2015, bien que la proportion d'incidence dans les communautés FAC ait diminué après trois cycles de dépistage actif, parallèlement à la prévalence stable dans les communautés FPC. Les NNS cumulatifs étaient de 34, 39 et 29 chez les personnes infectées par le VIH/SIDA, les personnes présentant des symptômes positifs de tuberculose et des antécédents de tuberculose, respectivement, comparativement à 1 478 pour le reste de la population. Le délai médian des patients sous ACF était d'un jour (intervalle interquartile, IQR : 0-27) par rapport au PCF de 30 jours (IQR : 14-61).
Conclusions: Cette étude a confirmé que l'ACF massif n'était pas efficace pour la population générale dans un contexte de prévalence modérée de la tuberculose. La priorité devrait être accordée à la définition et au ciblage des groupes à risque élevé dans la collectivité avant le lancement du processus de dépistage. L'intervalle de temps plus court entre l'apparition des symptômes de la tuberculose et l'établissement d'un lien avec les services de santé peut réduire le risque de transmission communautaire de la tuberculose. En outre, la stratégie intégrée d'ACF dans le projet national de santé publique élémentaire peut avoir un impact à long terme sur celle-ci.

Translated from English version into French by Yasmine Zongo, revised by Caroline Girard, through

TRANSLATORS WITHOUT BORDERS

Роль раннего выявления случаев заболевания при массовых обследованиях на туберкулез на уровне общин в китайской провинции Юньнань

Дзин-Оу Чень, Ю-Бинь Цю, Зульма Ванесса Руэда, Цзин-Лон Хоу, Кунь-Юнь Лу, Лю-Пин Чень, Вэй-Вэй Су, Ли Хуан, Фэй Чжао, Тао Ли, Линь Сюй

Реферат

Общие сведения: Препятствия для доступа к диагностике и лечению, в дополнение к недостаточной идентификации и регистрации случаев заболевания, приводят к распространению туберкулеза (ТБ) в населенных пунктах, особенно среди труднодоступных групп населения. В рамках данного исследования была проведена оценка стратегии раннего выявления случаев заболевания туберкулезом (РВЗ) на территориальном уровне среди групп высокого риска и населения в целом за период с 2013 по 2015 год.
Методы: это ретроспективное когортное исследование проводило РВЗ в десяти населенных пунктах округа Дунчуань, расположенных на северо-востоке провинции Юньнань за период с 2013 по 2015 год; и по сравнению с 136 населенными пунктами, в которых было пассивное выявление случаев заболевания (ПВЗ). Алгоритм РВЗ заключался в следующем: 1) скрининг на симптомы туберкулеза среди зарегистрированных в населенном пункте жителей путем посещений на дому, 2) пациентам с положительными симптомами, а также определенным группам высокого риска сделали рентген грудной клетки (CXR) с последующим подтверждением микроскопией мокроты. Пропорция заболеваемости туберкулезом и количество человек, которым необходим скрининг (NNS) для выявления одного случая, были рассчитаны для оценки стратегии РВЗ по сравнению с ПВЗ, критерий хи-квадрат применялся для сравнения доли заболеваемости в популяции больных туберкулезом и характеристик выявленных случаев в рамках различных стратегий. После этого для сравнения доли заболеваемости между населением в целом

и группами высокого риска применялись коэффициент заболеваемости (IRR) и точный тест Фишера по нескольким показателям. Пациенты и диагностические задержки для РВЗ и ПВЗ сравнивались с помощью критерия суммы рангов Уилкоксона.
Результаты: В общей сложности для проведения РВЗ были осмотрены в совокупности 97 521 зарегистрированный житель, 12,3 % из них были определены как группы высокого риска или имели симптомы туберкулеза. Шестьдесят шесть новых больных туберкулезом были выявлены с помощью РВЗ. Между совокупной долей заболеваемости ТБ в РВЗ (67,7/100 000 человек) и распространенностью заболевания в рамках ПВЗ (62,6/100 000 человек) в 2013-2015 годах существенных различий не было, хотя после трех раундов активного скрининга доля заболеваемости в населенных пунктах, прошедших РВЗ, снизилась, при сохранении стабильной распространенности в населенных пунктах, проходивших ПВЗ. Совокупный показатель NNS составлял 34, 39 и 29 у лиц, инфицированных ВИЧ/СПИДом, людей с положительными симптомами ТБ и перенесших предыдущие заболевания ТБ, соответственно, по сравнению со 1478 среди населения в целом. Средняя задержка пациента при РВЗ была один день (межквартильный диапазон, МКД: 0-27) по сравнению с 30 днями для ПВЗ (МКД: 14-61).
Выводы: это исследование подтвердило, что массовые РВЗ не были эффективны среди населения в целом при умеренной распространенности туберкулеза. Приоритетной задачей должно быть определение и целевые мероприятия для групп высокого риска в населенном пункте до начала процесса скрининга. Более короткий промежуток времени при РВЗ между появлением симптомов ТБ и обращением в медицинские учреждения может снизить риск передачи ТБ между группами населения. Кроме того, интегрированная стратегия РВЗ в Национальном проекте базовой службы общественного здравоохранения может иметь долгосрочные последствия для общественного здравоохранения.

Translated from English version into Russian by Michael Orlov, revised by Irina Nosova, through

TRANSLATORS
WITHOUT BORDERS

El papel de búsqueda de casos activos, basados en la comunidad, para detectar la tuberculosis, en Yunnan, provincia de China.

Jin-Ou Chen, Yu-Bing Qiu, Zulma Vanessa Rueda, Jing-Long Hou, Kun-Yun Lu, Liu-Ping Chen, Wei-Wei Su, Li Huang, Fei Zhao, Tao Li, Lin Xu.

Resumen

Antecedentes: Los obstáculos para acceder a un diagnóstico y recibir tratamiento, sumado a la deficiencia en la identificación y notificación de casos, deriva en que la tuberculosis (TB) se propague dentro de las comunidades, especialmente entre poblaciones de difícil acceso. Este estudio evaluó la estrategia de detección de casos activos de tuberculosis, en una comunidad, en China, en los años desde el 2013 al 2015, entre grupos de alto riesgo y la población en general.
Métodos: Este estudio retrospectivo de la cohorte llevó a cabo una búsqueda activa de casos (ACF; *por sus siglas en inglés*), en diez comunidades del condado de Dongchuan, ubicadas en el noreste de la provincia de Yunnan, entre los años 2013 y 2015; y en comparación con 136 comunidades, las cuales tenían una búsqueda pasiva de casos (PCF; *por sus siglas en inglés*). El algoritmo para ACF fue: 1) detectar síntomas de tuberculosis, entre los residentes inscritos en la comunidad, por visitas domiciliarias, 2) aquellos con síntomas positivos, junto con grupos definidos de alto riesgo, sometidos a rayos

X de tórax (CXR), seguidos de una confirmación de microscopía de esputo. La proporción de incidencia de la tuberculosis y el número necesario, para detectar un caso, se calcularon para evaluar la estrategia de ACF, en comparación con PCF, se aplicó una prueba de chi-cuadrado, para comparar la proporción de incidencia de la demografía de casos de tuberculosis y las características de casos detectados bajo diferentes estrategias**.** A partir de entonces, se aplicó la relación de tasa de incidencia (IRR; *por sus siglas en inglés*) y la prueba exacta de Fisher múltiple, para comparar la proporción de incidencia entre la población en general y los grupos de alto riesgo. Se compararon los retrasos por la prueba de la suma de rango Wilcoxon, en los pacientes y diagnósticos para ACF y PCF.

Resultados: En total, se visitaron a 97 521 residentes inscritos, con ACF, de forma acumulativa, 12,3% se definieron como grupos de alto riesgo o tenían síntomas de tuberculosis. Se detectaron por medio de ACF sesenta y seis nuevos pacientes con tuberculosis. No hubo diferencia significativa, entre la proporción acumulada de incidencia de tuberculosis para ACF (67,7/100 000 habitantes) y la prevalencia de PCF (62,6/100 000 habitantes), durante 2013 a 2015, aunque la proporción en las comunidades del ACF disminuyó, después de tres rondas de cribado activo concurrente, con una prevalencia estable, en las comunidades de PCF. El NNS acumulado fue de 34, 39 y 29, en individuos infectados con VIH / SIDA, personas con síntomas positivos de TB y antecedentes de TB previa, respectivamente, en comparación con 1478 de la población en general. La media del retraso del paciente bajo ACF fue de un día (rango inter-cuartil, IQR: 0–27), en comparación con PCF, con 30 días (IQR: 14–61).

Conclusiones: El presente estudio confirmó que la ACF masiva no fue efectiva, en la población general, en un entorno de prevalencia de TB moderada. La prioridad debe ser la definición y focalización de los grupos de alto riesgo, en la comunidad, antes de que se inicie el proceso de selección. El intervalo de tiempo más corto de ACF, entre el inicio de los síntomas de TB y la vinculación con el servicio de atención médica, puede disminuir el riesgo de transmisión comunitaria de TB. Además, la estrategia integrada de ACF, en el Proyecto Nacional del Servicio Básico de Salud Pública puede tener un impacto, a largo plazo, en la salud pública.

Translated from English version into Spanish by Maia Avruj, revised by Maria Luz Puerta, through

TRANSLATORS
WITHOUT BORDERS
