# Supplementary material for: Role of community-based active case finding in screening tuberculosis in Yunnan province of China
Source: Infect Dis Poverty. 2019 Oct 29;8:92. doi: 10.1186/s40249-019-0602-0 (PMC6819334; doi:10.1186/s40249-019-0602-0)
Supplement: Supplementary file 2 — Additional file 2: Table S1. Incidence proportion of new TB cases and number needed to screen in high-risk groups of active case finding strategy in Yunnan, 2013–2015. [file 40249_2019_602_MOESM2_ESM.docx]

**Additional file 1**

**Table 1** Incidence proportion of new TB cases and number needed to screen in high-risk groups of active case finding strategy in Yunnan, 2013-2015

|  | 2013 | | |  |  | 2014 | | |  |  | 2015 | | |  |
| --- | --- | --- | --- | --- | --- | --- | --- | --- | --- | --- | --- | --- | --- | --- |
| Groups of population | Cases. *n*  (NO. of Enrolled residents) | TB incidence proportion^a^ | Pairwise  *χ^2^* tests^b^ | NNS^c^ |  | Cases. *n*  (NO. of Enrolled residents) | TB incidence proportion^a^ | Pairwise  *χ^2^* tests^b^ | NNS^c^ |  | Cases. *n*  (NO. of Enrolled residents) | TB incidence proportion^a^ | Pairwise  *χ^2^* tests^b^ | NNS^c^ |
| General population | 6 |  |  |  |  | 8 |  |  |  |  | 2 |  |  |  |
|  | (29217) | 20.5 | a | 4870 |  | (29212) | 27.4 | a | 3652 |  | (27085) | 7.4 | a | 13543 |
| Positive symptom and high-risk groups^d^ | 28 |  |  |  |  | 19 |  |  |  |  | 3 |  |  |  |
|  | (4203) | 666.2 | b | 150 |  | (4073) | 466.5 | bc | 214 |  | (3731) | 80.4 | a | 1244 |
| Positive symptom | 5 |  |  |  |  | 0 |  |  |  |  | 0 |  |  |  |
|  | (134) | 3731.3 | c | 27 |  | (14) | 0.0 | abc | - |  | (45) | 0.0 | a | - |
| High-risk groups^d^ | 27 |  |  |  |  | 19 |  |  |  |  | 3 |  |  |  |
|  | (4151) | 650.4 | b | 154 |  | (4068) | 467.1 | bc | 214 |  | (3724) | 80.6 | a | 1241 |
| Elderly | 22 |  |  |  |  | 18 |  |  |  |  | 2 |  |  |  |
|  | (3815) | 576.7 | b | 173 |  | (3786) | 475.4 | bc | 210 |  | (3416) | 58.5 | a | 1708 |
| Diabetes | 4 |  |  |  |  | 0 |  |  |  |  | 2 |  |  |  |
|  | (643) | 622.1 | b | 161 |  | (692) | 0.0 | ab | - |  | (642) | 311.5 | a | 321 |
| HIV/AIDS | 2 |  |  |  |  | 0 |  |  |  |  | 0 |  |  |  |
|  | (23) | 8695.7 | b | 12 |  | (25) | 0.0 | abc | - |  | (20) | 0.0 | a | - |
| Close contact | 0 |  |  |  |  | 0 |  |  |  |  | 1 |  |  |  |
|  | (37) | 0.0 | b | - |  | (49) | 0.0 | abc | - |  | (69) | 1449.3 | a | 69 |
| History of previous  TB case | 12 |  |  |  |  | 3 |  |  |  |  | 0 |  |  |  |
|  | (150) | 8000.0 | c | 13 |  | (140) | 2142.9 | c | 47 |  | (141) | 0.0 | a | - |

a: Tuberculosis incidence proportion=new TB cases/population in with high-risk*100 000

b: Pairwise *χ2* tests were summarized as compact letter display, the different letters represented statistically significant difference between groups

c: NNS=number needed to screen to detect one case

d: High-risk groups=Elderly, Diabetes, HIV/AIDS, close contact, and history of previous TB case (includes pulmonary and extrapulmonary TB).
